# KNOWLEDGE MANAGEMENT AND THE ADOPTION OF CULTURE CHANGE INITIATIVES BY HIGH-MEDICAID-CENSUS NURSING HOMES

**DOI:** 10.1093/geroni/igz038.2568

**Published:** 2019-11-08

**Authors:** Larry Hearld, Akbar Ghiasi, Jeffery Szychowski, Robert Weech-Maldonado

**Affiliations:** University of Alabama at Birmingham, Birmingham, Alabama, United States

## Abstract

Culture change represents an organizational transformational process to become person-centered, through staff and resident empowerment. Culture change initiatives have been associated with fewer health-related deficiency citations and better psychosocial outcomes. Knowledge management, defined as the process of creating or locating knowledge, and managing the dissemination of knowledge within and between organizations, has been shown to be associated with the adoption of innovations such as culture change initiatives. This study examines the relationship between knowledge management activities of high Medicaid census (70% or higher) nursing homes (NHs) and the adoption of culture change initiatives. This study used facility survey data from approximately 324 nursing home administrators (30% response rate) from 2017- 2018, merged with data from LTCFocus, Area Health Resource File, and Medicare Cost Reports. Binary logistic regression models revealed that the probability of adopting a culture change initiative was 0.12 higher for facilities reporting a one-unit higher level of knowledge management activities. Additional interaction analysis revealed that knowledge management activities were associated with a greater likelihood of adopting a culture change initiative for NHs where the director had been in his/her position fewer years. Similarly, higher levels of overall knowledge management activities were significantly associated with greater adoption of culture change initiatives at intermediate levels of nurse retention. Results suggest that knowledge management activities may help high Medicaid NHs acquire and mobilize informational resources in ways that can support the adoption of patient-centered initiatives. These activities may be particularly effective in nursing homes with leadership and nursing staff instability.

